# 827. Adjunct terbinafine in patients with leukemia and invasive fusariosis with skin lesions: Discordance between responses of skin lesions and systemic outcomes

**DOI:** 10.1093/ofid/ofad500.872

**Published:** 2023-11-27

**Authors:** Takahiro Matsuo, Sebastian Wurster, Ying Jiang, Jeffrey Tarrand, Dimitrios P Kontoyiannis

**Affiliations:** The University of Texas MD Anderson Cancer Center, Houston, TX; The University of Texas MD Anderson Cancer Center, Houston, TX; The University of Texas MD Anderson Cancer Center, Houston, TX; The University of Texas MD Anderson Cancer Center, Houston, TX; The University of Texas MD Anderson Cancer Center, Houston, TX

## Abstract

**Background:**

Invasive fusariosis (IF) is a severe opportunistic mold infection with poor outcomes in immunosuppressed leukemia patients (pts). Skin involvement is the second most common manifestation of IF after pneumonia. While *in vitro* studies have shown synergy of terbinafine (TRB) in combination with triazoles or liposomal amphotericin B (L-AMB) for a variety of fungi, including Fusarium species, clinical data are sparse.

**Methods:**

We retrospectively reviewed adult leukemia pts with proven or probable IF at MD Anderson Cancer Center (Houston, TX) between 2009 and 2021 who received adjunct TRB (aTRB) for skin manifestations of IF, in view of its high bioavailability in skin and skin structures. Demographics, clinical manifestations, antifungal therapy, and outcomes were reviewed.

**Results:**

Among 140 IF pts, we identified 15 pts who received aTRB (dose range: oral 250-1,000 mg/day, duration range: 3-128 days; topical cream in 1 pt) for IF with skin involvement (Table 1). Their median age was 50 (range 21-80); 14 (93%) were male. Eleven pts (73%) had acute myeloid leukemia/myelodysplastic syndrome, 14 (93%) had neutropenia (< 500/µL), and 5 (33%) had a prior hematopoietic stem cell transplant. 13 pts (87%) had disseminated skin lesions, 1 had a localized skin lesion, and 1 had onychomycosis. All 15 patients received antifungal combination therapy. In addition to aTRB, 13 pts received voriconazole + L-AMB, 1 received posaconazole + L-AMB, and 1 received isavuconazole. The median time from IF diagnosis to aTRB initiation was 8 days (range: 0-74). While skin lesions on day 42 after aTRB initiation or at the time of earlier death were improved (6 pts, 40%) or stabilized (4 pts, 27%) in two thirds of patients, the same proportion of pts (10/15, 67%) had progression of IF in sites with poor pharmacologic exposures of TRB (lungs in 7 pts, sinus in 2 pts, endophthalmitis in 1 pt). 10 out of 15 pts died by day 42 (67%) after aTRB initiation.

Patient demographics and treatment outcome

**Figure:**
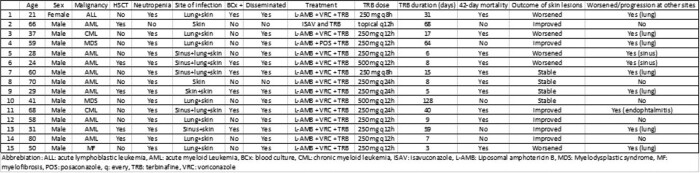

**Table 1.**

**Conclusion:**

In our limited series, aTRB seemed to display site (skin only)-specific synergistic activity with other systemic antifungals in IF, which is consistent with in vitro synergistic effects and pharmacokinetic aspects of this allylamine which has high skin concentrations. However, TRB use did not affect IF outcomes that were poor in the setting of persistent immunosuppression.

**Disclosures:**

**Dimitrios P. Kontoyiannis, MD, MS, ScD, PhD**, AbbVie: Board Member|Astellas: Grant/Research Support|Cidara: Board Member|Gilead: Grant/Research Support|Merck: Advisor/Consultant|Scynexis/MSGERC: Board Member

